# Long-term clinical and radiographic outcomes after locking plate fixation of proximal humerus fractures: a 6-year follow-up study

**DOI:** 10.1016/j.jseint.2026.101683

**Published:** 2026-03-05

**Authors:** Jan Theopold, Tobias Schöbel, Yasmin Youssef, Pierre Hepp

**Affiliations:** Division of Arthroscopic and Special Joint Surgery/Sports Injuries, Department of Orthopedics, Trauma and Plastic Surgery, University of Leipzig, Leipzig, Germany

**Keywords:** Proximal humerus fractures, Locking plate fixation, Avascular necrosis, Post-traumatic osteoarthritis, Long-term outcome, Constant score, Functional recovery, Complication rate

## Abstract

**Background:**

Locking plate fixation is widely used for the treatment of displaced proximal humerus fractures; however, robust long-term data regarding its function and late complications remain limited. We report 6-year clinical and radiographic outcomes of locking plate fixation, including data regarding the relationship between avascular necrosis (AVN), post-traumatic osteoarthritis (PTA), and shoulder function.

**Methods:**

Between 2017 and 2019, 31 consecutive patients (21 women, 10 men; mean age, 63 years) with displaced proximal humerus fractures underwent fixation with a locking plate. Follow-up data were obtained at 3, 12, and 72 months. The fractures were classified using the Neer classification system (9 patients with 2-part fractures, 16 with 3-part fractures, and 6 with 4-part fractures). Functional outcomes were assessed using the Constant score. Radiographs were evaluated for AVN (y/n) and PTA (y/n). Reoperations and complications were also recorded.

**Results:**

At 72 months, the mean Constant score was 66.2, with no improvement beyond 12 months. AVN occurred in 19% of patients and PTA in 34% of patients. Despite marked radiographic changes, the patients with AVN showed better shoulder function than those with PTA (*P* < .05). Reoperations were required in 32% of patients at a mean of 11 months and were most commonly implant removal with or without arthrolysis.

**Conclusion:**

Long-term function after locking plate fixation is moderate and plateaus after one year. Although radiographically severe, AVN does not necessarily impair shoulder function. In contrast, PTA is associated with progressive functional decline. These findings emphasize the importance of differentiating between necrotic and degenerative radiographic sequelae and of carefully tailoring the indication for locking plate fixation, particularly in complex fracture patterns.

Proximal humerus fractures (PHFs) are among the most common fractures in the elderly population and continue to present a therapeutic challenge, particularly in osteoporotic bones.^4^^,^^6^^,^^13^ With demographic changes of an aging population, their sociodemographic relevance is constantly rising. PHFs represent the third most common age-related fracture in the elderly population, with women more frequently affected than men. Past studies from the inpatient sector have reported rates ranging from 60.1 to 90.8 per 100,000 person-years.^9^^,^^17^ However, a current study that has evaluated outpatient data showed that the actual incidence of PHFs is approximately 1.7 times higher than previously assumed.^11^

Locking plate fixation has been used to restore anatomy and allow early mobilization. However, especially in osteoporotic bone, complications include loss of reduction, screw cutout, and fixation failure.^10^ Furthermore, the long-term durability of outcomes after locking plate fixation remains uncertain due to the risk of late sequelae, including avascular necrosis (AVN) and post-traumatic osteoarthritis (PTA). Although several studies have documented short- and mid-term results, there remains a paucity of long-term data exceeding five years of follow-up.^1^^,^^12^^,^^14^ The reported incidence of AVN varies widely across studies, and its impact on shoulder function remains a subject of debate. Recent studies have indicated that AVN does not uniformly predict poor functional outcomes, whereas degenerative sequelae appear to be more detrimental for shoulder mobility and patient-reported outcomes.^3^^,^^20^

We aimed to assess the long-term clinical and radiographic outcomes following locking plate fixation of displaced PHFs and to analyze the relationship between AVN, PTA, and functional outcome.

We hypothesized that long-term functional outcome after locking plate fixation of PHFs differs between patients who develop AVN and those who develop PTA.

## Materials and methods

This single-center, prospective, nonrandomized study included 31 patients treated between 2017 and 2019 for displaced PHFs using locking plate fixation at the proximal humerus (WinstaPH; Fa Axomed Spaichingen, Germany).

The study was conducted in accordance with the Declaration of Helsinki and good clinical practice guidelines and approved by the local institutional ethics committee (ethics approval number 0022/17-ek). Written informed consent was obtained from all participants prior to inclusion in the study.

### Inclusion and exclusion criteria

Patients were eligible for inclusion if they presented with a displaced PHF classified as a 2-, 3-, or 4-part fracture according to the Neer classification and were treated primarily with a locking plate. According to the Neer classification, type II fractures represented surgical neck fractures, while type IV fractures were classified as true 4-part fractures. No further subclassification was applied. Additional inclusion criteria comprised an age of 18 years or older, availability for clinical and radiographic follow-up at 3, 12, and 72 months, and provision of written informed consent. Patients were excluded in the presence of pathological fractures (eg, metastatic or metabolic bone disease). Further exclusion criteria included polytrauma that precluded standardized postoperative rehabilitation, cognitive or neurological impairment interfering with follow-up or functional testing, and incomplete data or loss to follow-up.

Of the original study cohort, 31 patients were available for final long-term analysis. Two patients were excluded due to incomplete documentation at long-term follow-up. Only patients with complete clinical and radiographic datasets were included in the final evaluation.

**Figure 1:**
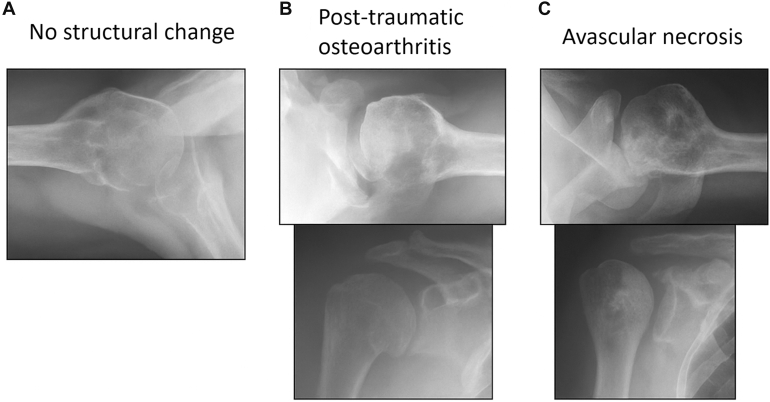
Representative radiographs showing typical long-term outcomes following locking plate fixation of proximal humerus fractures. (**A**) No structural change; (**B**) development of post-traumatic osteoarthritis with joint-space narrowing and osteophyte formation; (**C**) avascular necrosis with segmental collapse of the humeral head.

Clinical and radiographic follow-up examinations were performed at 3, 12, and 72 months. Shoulder function was evaluated using the Constant score (CS) (Fig. 1).^5^

AVN and PTA were assessed on standard radiographs and recorded as a binary variable (present vs. absent). No formal classification or grading of AVN severity was applied. Complications and reoperations were documented.

Data were collected using CTX Excel (Microsoft Corporation, Redmond, WA, USA) for descriptive analyses. All statistical analyses were performed using SPSS V28.0 (IBM, Armonk, NY, USA). Statistical comparisons were performed between subgroups using *t*-tests, with significance set at *P* < .05.

## Results

**Figure 2:**
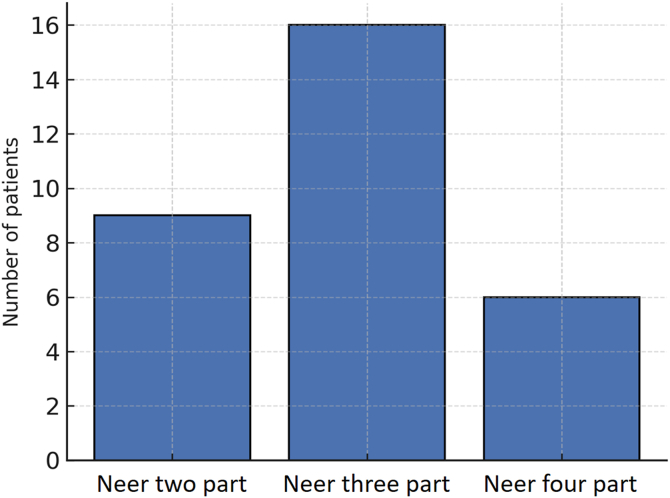
Fracture pattern distribution according to Neer classification. Most fractures were 3-part (n = 16), followed by 2-part (n = 9) and 4-part (n = 6) configurations.

The patients included 21 women and 10 men with a mean age of 63 years (range, 42-81 years). Fractures were classified according to the Neer classification system as follows: 9 patients with 2-part fractures, 16 with 3-part fractures, and 6 with 4-part fractures (Fig. 2).

The mean CS increased from 38.4 ± 11.2 at 3 months to 65.1 ± 12.7 at 12 months, reaching 66.2 ± 13.5 at 72 months. A significant improvement was observed during the first post-operative year (*P* < .001), but further improvement was not significant.

**Figure 3:**
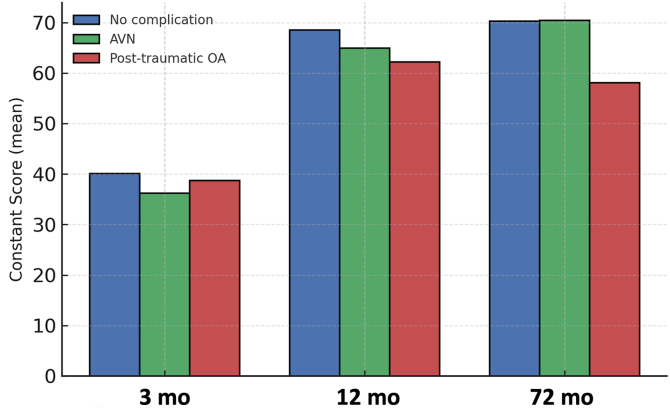
Functional outcome over time following locking plate fixation (Constant score development). The mean Constant score improved between 3 and 12 months, then stabilized. Patients with avascular necrosis maintained satisfactory function, whereas those with post-traumatic osteoarthritis showed late decline. *AVN*, avascular necrosis; *OA*, osteoarthritis.

In the subgroup analysis, patients without radiographic complications achieved mean CS values of 40.1, 68.5, and 70.3 at 3, 12, and 72 months. Those with AVN (n = 6) improved from 36.2 to 64.9 and 70.4, while patients with PTA (n = 10) improved from 38.7 to 62.2 but declined to 58.1 at the final follow-up. At 72 months, patients with AVN had significantly better functioning than those with PTA (mean difference 12.3 points; *P* = .03). AVN was observed predominantly in patients with more complex fracture patterns, whereas PTA occurred across different fracture types without a clear pattern-specific association. AVN was identified in 6 patients (19%), all of whom had Neer 4-part fractures, whereas PTA developed in 10 patients (34%) across different fracture types (Fig. 3).

Ten patients (32%) required reoperation after a mean of 11 months (range, 6-22 months). Procedures included implant removal (n = 3), partial removal (n = 2), and arthrolysis (n = 5). No infections or implant failures occurred beyond the first year (Table I). At final follow-up, no additional revision procedures were requested or performed beyond the secondary interventions already documented during the postoperative course.

**Table I:** Reoperations after locking plate fixation of proximal humerus fractures.

Type of reoperation	Patients (n)	Percent (%)
None	21	67.7
Implant removal	3	9.7
Partial implant removal	2	6.5
Removal + arthrolysis	5	16.1

Secondary procedures, most frequently implant removal combined with arthrolysis within the first postoperative year, were required in 32% of patients.

Overall shoulder function improved after surgery, as demonstrated by an increase in the CS from early post-operative follow-up to final long-term assessment.

Secondary procedures, primarily implant removal with or without arthrolysis, led to subjective improvement of symptoms and shoulder mobility in most cases; however, no systematic reassessment of functional outcome after revision surgery was performed.

## Discussion

This prospective 6-year follow-up study demonstrates that functional recovery after locking plate fixation of displaced PHFs occurs mainly within the first post-operative year and remains stable thereafter. Beyond this period, shoulder function remains stable. Early post-operative outcome, therefore, appears to be a reliable predictor of long-term shoulder function. The results of the current study confirm that AVN of the humeral head, although radiographically severe, does not necessarily correspond to clinical failure. Patients with AVN demonstrated functional outcomes comparable to those without radiographic complications and superior to those with PTA. This finding aligns with recent studies suggesting that partial necrosis may be remodeled into a stable and pain-free configuration.^7^^,^^14^^,^^19^

Conversely, PTA is a chronic degenerative process that leads to capsular stiffness, pain, and progressive limitation of motion.^16^ In this series, PTA was associated with a decline in the CS after one year, emphasizing its negative impact on long-term outcomes.^2^^,^^8^ Taken together, the data indicate that AVN and PTA represent distinct radiographic sequelae with different implications for long-term shoulder function.

The reoperation rate of 32% in this study, primarily for stiffness or hardware irritation, is consistent with that reported in other long-term studies.^12^^,^^15^ The risk factors for AVN, fracture complexity, and medial hinge disruption have been confirmed, with a higher incidence in patients with 4-part fractures.^3^^,^^18^ This finding should be interpreted descriptively and may reflect the higher initial injury severity associated with 4-part fractures rather than a causal relationship.

This study was limited by its modest sample size and absence of a nonoperative control group. Radiographic analysis was limited to plain radiographs, which may have underestimated subtle degenerative changes.

Due to the long study period, the total number of PHFs treated and the exact number of involved surgeons could not be reliably reconstructed, introducing a potential selection bias.

Accordingly, the present analysis does not allow differentiation between early and advanced stages of AVN or PTA, including the extent of humeral head collapse or joint space narrowing.

The adequacy of fracture reduction and implant positioning was not formally graded or quantified and therefore could not be analyzed in relation to the development of AVN or PTA.

Nevertheless, the prospective design, standardized follow-up, and 6-year observation period provide valuable insights into the long-term behavior of plated PHFs. However, even within the limitations of this study, the observed differences between AVN and PTA highlight distinct long-term functional trajectories.

## Conclusion

At 6 years of follow-up, locking plate fixation as an option for treatment of PHFs provides moderate but durable functional results. Although AVN does not impair shoulder function, PTA leads to progressive worsening. Therefore, surgeons should differentiate between necrotic and degenerative sequelae when interpreting long-term outcomes and selecting candidates for surgery.

## Disclaimers

Funding: No funding was disclosed by the authors.

Conflicts of interest: The authors, their immediate families, and any research foundations with which they are affiliated have not received any financial payments or other benefits from any commercial entity related to the subject of this article.

## Declaration of AI and AI-assisted technologies in the writing process

During the preparation of this work, the author(s) used Deepl and LanguageTool in order to translate/rephrase the manuscript. After using this tool/service, the author(s) reviewed and edited the content as needed and take(s) full responsibility for the content of the published article.
